# Editorial: Update on the Treatment of Metastatic Non-small Cell Lung Cancer (NSCLC) in New Era of Personalised Medicine

**DOI:** 10.3389/fonc.2017.00311

**Published:** 2017-12-13

**Authors:** Barbara Melosky

**Affiliations:** ^1^University of British Columbia, Vancouver, BC, Canada

**Keywords:** NSCLC, advanced, targeted therapy, immunotherapy, editorial

**Editorial on the Research Topic**

**Update on the Treatment of Metastatic Non-small Cell Lung Cancer (NSCLC) in New Era of Personalised Medicine**

We are honored and privileged to present 11 review articles in the context of “An Update on the Treatment of Non Squamous Non-small Cell Lung Carcinoma (NSCLC) in the era of Personalized Medicine.” Gone are the days when all lung cancers are treated the same. Treatment is now personalized for multiple biomarkers leading to major advances in survival and quality of life.

It has been over a decade since the IPASS trial was published showing us that patients with an EGFR mutation fare better with a first-generation EFGR TKI targeted to inhibit that mutation versus chemotherapy. In this update, results of clinical trials of the second-generation EGFR inhibitor, afatinib are explored (Morin-Ben Abdallah and Hirsh). The benefit in treatment naïve, refractory, and squamous histology is reviewed reflecting a benefit of irreversibly inhibiting all ErbB family members. As we identify resistance mechanisms to both first- and second-generation EGFR TKI’s, the use and benefit of third-generation inhibitors has become standard of care (Barnes et al.). The treatment paradigm of ALK rearrangement is rapidly changing. This incredible journey is leading to prolonged survival in these patients. With next generation sequencing, rare oncogenic drivers are found and successful drug development has occurred (Daoud and Chu). Histology is itself a biomarker and with multiple mutations now being found, the practicing oncologist is challenged. A series of algorithms will be presented for non-squamous histology (Melosky). Squamous histology deserves its own attention and potential molecular targets and novel agents are explored (Soldera and Leighl).

You cannot mention advances in lung cancer without discussing the amazing story of immune-oncology. Activating one’s own immune system has led to impressive survival in thoracic malignancies. Future investigations combining PD-1/L1 with chemotherapy, targeted therapy, or other immune-oncology agents aim to improve the number of patients to benefit are ongoing (Iafolla and Juergens).

Targeting angiogenesis is recognized as an effective treatment strategy in a multitude of malignancies including lung cancer. Adding angiogenesis inhibitors to EGFR inhibitors has promising results. Preclinical evidence suggesting an immunosuppressive effect of pro-angiogenic factors leads also to the rationale of adding these agents to immune checkpoint inhibitors (Tabchi and Blais).

Addressing and maintaining quality of life has always been on the forefront goal of lung oncologists. Because of this and impact on lifespan, brain metastases have been an important issue to address. Both targeted therapy and immunotherapy have changed the natural history and our treatment in this important metastatic site (Wong). Thoracic oncologists have had to become experts in systemic therapy of pain. The field of interventional pain management needs to be highlighted as it leads to comprehensive patient care (Morin-Ben Abdallah and Hirsh).

Finally, carcinoid and atypical carcinoid tumors of lung origin are reviewed as new treatment options exist and education in these rare tumors is lacking (Melosky).

As far as we have come in the past few years, we still have to strive for improvement in patients with advanced disease. Molecular testing for should be standardized. Patients with non-squamous histology and never smokers should have molecular testing to include at least EGFR, ALK, ROS 1, and BRAF. In addition, genes that could and should be tested include RET, HER2, NTRK, and C MET Exon 14 Skip. Driver mutations continue to be discovered and therapeutics directed toward them continue to be developed. We should not stop trying to inhibit KRAS, the most prevalent mutation in adenocarcinoma. Patients with advanced disease squamous histology should also be tested with molecular panels. CMET Exon 14 Skip is just one example of a driver in squamous histology that when identified may be treated appropriately. PD-L1 testing should be done reflexively on all patients. Although immuno-oncology has changed the treatment landscape, many still do not benefit or respond. Combination therapy may be the answer but toxicity must be low. Our goal for our patients it to prolong their survival duration while maintaining a high quality of their life.

We welcome you to read this issue. The treatment has become complex but the winner is our patients who are now living longer and sustaining a higher quality of life as their treatment is now focused on their tumor factors. The world of lung cancer has changed dramatically with personalized medicine.

## Author Contributions

The author confirms being the sole contributor of this work and approved it for publication.

## Conflict of Interest Statement

The author declares that the research was conducted in the absence of any commercial or financial relationships that could be construed as a potential conflict of interest.

